# A survey on the availability of geriatric-friendly protocols, equipment and physical environment across emergency departments in Flanders, Belgium

**DOI:** 10.1186/s12877-023-03994-z

**Published:** 2023-05-03

**Authors:** Pieter Heeren, Lotte Lombaert, Petra Janssens, Farah Islam, Johan Flamaing, Marc Sabbe, Koen Milisen

**Affiliations:** 1 Department of Public Health and Primary Care, Academic Centre for Nursing and Midwifery, KU Leuven, Kapucijnenvoer 35/4, 3000 Leuven, Belgium; 2 Department of Geriatric Medicine, University Hospitals Leuven, Herestraat 49, 3000 Leuven, Belgium; 3 Research Foundation Flanders, Egmontstraat 5, 1000 Brussels, Belgium; 4 Department of Public Health and Primary Care, Gerontology and Geriatrics, KU Leuven, Herestraat 49, 3000 Leuven, Belgium; 5 Department of Emergency Medicine, University Hospitals Leuven, Herestraat 49, 3000 Leuven, Belgium; 6 Department of Public Health and Primary Care, Emergency Medicine, KU Leuven, Kapucijnenvoer 35/4, 3000 Leuven, Belgium

**Keywords:** Geriatric emergency medicine, Acute care, Emergency department, Older adults

## Abstract

**Background:**

The acquisition of geriatric-friendly resources is an important part of adapting emergency department (ED) care to the needs of vulnerable older patients. The aim of this study was to explore the availability of geriatric-friendly protocols, equipment and physical environment criteria in EDs and to identify related improvement opportunities.

**Methods:**

The head nurse of 63 EDs in Flanders and Brussels Capital Region was invited to complete a survey in collaboration with the chief physician of the ED. The questionnaire was inspired by the American College of Emergency Physicians Geriatric ED Accreditation Program and explored the availability, relevance and feasibility of geriatric-friendly protocols, equipment and physical environment. Descriptive analyses were performed. A region-wide improvement opportunity was defined as a resource that was never to occasionally (0–50%) available on Flemish EDs and was scored (rather or very) relevant by at least 75% of respondents.

**Results:**

A total of 32 questionnaires were analysed. The response rate was 50.8%. All surveyed resources were available in at least one ED. Eighteen out of 52 resources (34.6%) were available in more than half of EDs. Ten region-wide improvement opportunities were identified. These comprised seven protocols and three physical environment characteristics: 1) a geriatric approach initiated from physical triage, 2) elder abuse, 3) discharge to residential facility, 4) frequent geriatric pathologies, 5) access to geriatric specific follow-up clinics, 6) medication reconciliation, 7) minimising ‘nihil per os’ designation, 8) a large-face, analogue clock in each patient room, 9) raised toilet seats and 10) non-slip floors.

**Conclusions:**

Currently available resources supporting optimal ED care for older patients in Flanders are very heterogeneous. Researchers, clinicians and policy makers need to define which geriatric-friendly protocols, equipment and physical environment criteria should become region-wide minimum operational standards. Findings of this study are relevant to facilitate the development process of this endeavour.

**Supplementary Information:**

The online version contains supplementary material available at 10.1186/s12877-023-03994-z.

## Background

Older adults presenting to the emergency department (ED) are characterized by interacting multi-domain problems and outcomes that are generally poorer compared to those of their younger counterparts [1–4]. As complaint-oriented approaches in EDs have shown to be suboptimal to manage this vulnerable subgroup of the ED population, geriatric emergency guidelines were developed [5, 6]. To facilitate its integration in clinical care, the American College of Emergency Physicians (ACEP) launched in 2018 the Geriatric ED Accreditation Program, which focusses on seven domains (i.e. staffing, education, policies/protocols/guidelines/procedures, quality improvement, outcome measurement, equipment/supplies, physical environment) and differentiates between EDs that deliver basic, advanced and high-advanced geriatric care [7]. A particular strength of this framework is that it is the first initiative introducing priorities for the integration of the numerous geriatric emergency care recommendations prompted in the existing literature [8].

In Belgium, the ED population is growing and aging. The total number of admissions to Belgian EDs increased by 23% between 2010 and 2019 (from 3,038,909 to 3,737,898 ED admissions), while over the same period of time the relative growth in the number of ED patients over 65 and over 75 years old was 38.5% and 30.7%, respectively. In 2019, the proportion of ED patients over 65 and 75 years old was 20% and 12%, respectively [9]. Although Belgian EDs have no legal obligation to adapt their care processes to accommodate the needs of older patients and no regional quality improvement initiatives have been established, a survey from 2014 reported that the majority of these EDs had already set up collaborations with geriatric departments [10]. However, this study mainly focused on the access to geriatric practitioners (e.g. geriatricians and members of the inpatient geriatric consultation team) in Belgian EDs. Therefore, little is known about which geriatric-friendly resources (i.e. protocols, equipment and physical environment criteria) are available on these EDs to support optimal care for older patients.

## Methods

### Aim

The aim of this study was to explore the availability of geriatric-friendly protocols, equipment and physical environment criteria in EDs and to identify related improvement opportunities.

### Study design

A cross-sectional survey was performed in Belgian EDs with Dutch as working language. This concerned a total of 63 EDs, which were all located in Flanders (i.e. the northern part of Belgium), except for one. One ED in the bilingual Brussels Capital Region (French and Dutch) was eligible, as well. As the questionnaire was only available in Dutch, EDs with French as working language were not approached for study participation. These non-eligible EDs were situated in the southern part of Belgium (i.e. Wallonia) and in the bilingual Brussels Capital Region. This manuscript was reported using the Consensus-Based Checklist for Reporting of Survey Studies (CROSS) (see Additional file 1) [11].

### Participants

All head nurses in the membership database of the Emergency Nurses Association in Flanders were invited to complete a questionnaire in collaboration with the chief physician of the ED.

### Questionnaire

A four-part questionnaire was developed based on the ACEP Geriatric ED Accreditation Program and the questionnaire of a previous survey on geriatric support in Belgian EDs [7, 10]. Part one, two and three of the questionnaire focussed on sample characteristics, general views on geriatric emergency care and the availability of geriatric-skilled staff, respectively. In part four, operational standards regarding protocols, equipment and physical environment were scored according three criteria: availability, relevance and feasibility. The availability of each standard was dichotomously (yes or no) operationalised using following question: “Does current practice correspond to this standard?” Both relevance and feasibility were measured using a four-point Likert scale (i.e. not…/rather not…/rather…/very…). This manuscript only focusses on data from part one and four of the questionnaire.

The questionnaire was developed in two phases. The first phase consisted of three rounds in which the senior authors (JF, MS and KM) gave feedback on the readability, comprehensibility and completeness of a draft version developed by the junior authors (LL, PJ and PH). Subsequently, in the second phase, the board of the Flemish Emergency Nurses Association assessed the readability, comprehensibility and completeness of the questionnaire. The final questionnaire is available as Additional file 2.

### Data collection

Data were collected from 9 January 2020 until 13 March 2020 using an online platform, Qualtrics [12]. After invitations for study participation were sent by the Emergency Nurses Association in Flanders, three follow-up initiatives were taken to stimulate the response rate. The Emergency Nurses Association in Flanders sent eligible participants an electronic reminder by e-mail at two time points (i.e. at one month and two months after onset of data gathering). Furthermore, in February 2020, the research team contacted all eligible participants by telephone to clarify the rationale of the study and seek engagement for study participation.

### Data analysis

Statistical Package for the Social Sciences (version 25) and Excel 2016 were used to calculate frequencies, modes, medians, means, standard deviations, quartiles and interquartile ranges (IQR), as appropriate. Questionnaires with a missing data rate of 50% or more were excluded from analysis. Availability of standards was classified into five categories: never (0%), seldom (1–25%), occasionally (26–50%), often (51%-75%) and very often (76–100%). ‘*Region-wide improvement opportunities*’ were defined as resources that were never to occasionally (0–50%) available, while being scored (rather or very) relevant by at least 75% of respondents. These region-wide improvement opportunities were classified as ‘difficult to achieve’ and ‘easy to achieve’ if at least half of respondents scored the operational standard ‘not (or rather not) feasible’ and ‘(rather or very) feasible’, respectively.

## Results

### Sample

Qualtrics registered 61 questionnaires. Of these, four overlapped with other registrations. For example, two head nurses of one ED started completing the questionnaire separately, and in three other cases, the head nurse interrupted completion of the questionnaire and began all over in a new questionnaire. These incomplete and initial registrations were excluded. Therefore, the number of different responses received was 57. As one respondent withdrew consent for study participation after questionnaire completion and as 24 questionnaires had a missing data rate of 50% or more, the final data analyses were based on 32 questionnaires, which implies a response rate of 50.8% (≈32/63). The final data analyses represented 32 EDs, located in three university and 28 non-university hospitals. Head nurses (*n* = 32) had a male/female ratio of 69:31, a median age of 49 years old (IQR = 44–56) and a median seniority in the current job of 12 years (IQR = 5–15). Chief ED physicians (*n* = 32) had a male/female ratio of 56:44, a median age of 48 years old (IQR = 42–55) and a median seniority in the current job of 7 years (IQR = 7–13).

### Protocols

Out of 27 surveyed geriatric-friendly protocols, one ED (3.1%) reported to have none, while another (3.1%) had 22. The majority of respondents (*n* = 21; 65.6%) reported having between 1 to 9 protocols. Nine EDs (i.e. 28.1%) had between 10 to 19 protocols. Table 1 gives an overview of frequent available protocols and protocols that were identified ‘region-wide improvement opportunities’. Additional file 3 reports few available protocols that were not identified ‘region-wide improvement opportunities’.

**Table 1:** Availability, relevance and feasibility of frequently available protocols and protocols that were identified ‘region-wide improvement opportunities’

	**Does current practice correspond to this standard?** **Rather relevant** **n (%)**		**How relevant is this standard?** n (%)				**How feasible is this standard?** n (%)			
			**Not relevant**	**Rather not relevant**	**Rather relevant**	**Very relevant**	**Not feasible**	**Rather not feasible**	**Rather feasible**	**Very feasible**
	**Yes**	**No**								
A guideline for **general practitioner notification**	25 (78,1)	7 (21,9)	0 (0)	0 (0)	13 (40,6)	19 (59,4)	0 (0)	0 (0)	16 (50)	16 (50)
A guideline to **minimize use of physical restraints**	23 (71,9)	9 (28,1)	0 (0)	1 (3,1)	14 (43,8)	17 (53,1)	0 (0)	3 (9,4)	16 (50)	13 (40,6)
A guideline for **standardized fall assessment** guideline with appropriate follow-up	22 (68,8)	10 (31,3)	1 (3,1)	3 (9,4)	19 (59,4)	9 (28,1)	1 (3,1)	7 (21,9)	17 (53,1)	7 (21,9)
Access to **transportation services** for return to residence	20 (62,5)	12 (37,5)	0 (0)	2 (6,3)	16 (50)	14 (43,8)	2 (6,3)	6 (18,8)	12 (37,5)	12 (37,5)
A guideline for **pain control** in elder patients	18 (56,3)	14 (43,8)	2 (6,3)	2 (6,3)	10 (31,3)	18 (56,3)	1 (3,1)	4 (12,5)	14 (43,8)	13 (40,6)
A guideline to standardize and minimize **urinary catheter use**	17 (53,1)	15 (46,9)	0 (0)	0 (0)	20 (62,5)	12 (37,5)	0 (0)	3 (9,4)	18 (56,3)	11 (34,4)
A guideline to define **criteria** for access to **Geriatric Emergency Department Care from ED triage**	8 (25)	24 (75)	2 (6,3)	4 (12,5)	20 (62,5)	6 (18,8)	4 (12,5)	11 (34,4)	14 (43,8)	3 (9,4)
A guideline for identification of **elder abuse** with appropriate follow-up	7 (21,9)	25 (78,1)	0 (0)	6 (18,8)	21 (65,6)	5 (15,6)	2 (6,3)	10 (31,3)	16 (50)	4 (12,5)
A guideline to address **transitions of care to residential care**	13 (40,6)	19 (59,4)	1 (3,1)	4 (12,5)	10 (31,3)	17 (53,1)	0 (0)	8 (25)	13 (40,6)	11 (34,4)
A **protocol** for the **work-up and initial treatment** of at least three **frequently occurring ED presentations** in older patients	13 (40,6)	19 (59,4)	0 (0)	2 (6,3)	17 (53,1)	13 (40,6)	0 (0)	5 (15,6)	17 (53,1)	10 (31,3)
Standardized **access to geriatric specific follow-up clinics** (e.g. geriatric assessment clinic, falls clinic, memory clinic)	9 (29,0)^a^	22 (71,0)^a^	6 (18,8)	2 (6,3)	16 (50)	8 (25)	7 (21,9)	6 (18,8)	14 (43,8)	5 (15,6)
A guideline for **medication reconciliation** in conjunction with a pharmacist	5 (15,6)	27 (84,4)	2 (6,3)	4 (12,5)	13 (40,6)	13 (40,6)	4 (12,5)	17 (53,1)	6 (18,8)	5 (15,6)
A guideline to **minimize nihil per os designation** and to promote access to appropriate food and drink	5 (16,1)^a^	26 (83,9)^a^	3 (9,4)	3 (9,4)	15 (46,9)	11 (34,4)	2 (6,3)	16 (50)	8 (25)	6 (18,8)

^a^(*n* = 31)

One protocol was very often available in Flemish EDs. This comprised a protocol to inform a patient’s general practitioner after ED discharge (*n* = 25/32; 78.1%). Often available protocols focused on physical restraint use (*n* = 23/32; 71.9%), fall risk assessment (*n* = 22/32; 68.8%), access to patient transport services (*n* = 20/32; 62.5%), pain management (*n* = 18/32; 56.3%), and urinary catheter use (*n* = 17/32; 53.1%). Five easy to achieve region-wide improvement opportunities were also identified. These included i) a protocol with criteria for access to a geriatric approach starting from physical triage, ii) an elder abuse protocol, iii) a protocol to facilitate discharge to a residential facility, iv) protocols for work-up and initial treatment of frequent geriatric pathologies and v) a protocol for access to geriatric-specific follow-up clinics. Two protocols were identified as difficult to achieve region-wide improvement opportunities. These focused on medication reconciliation and minimisation of ‘nihil per os’ status including better access to appropriate drinks and food.

### Equipment

Out of 14 surveyed elements, 14 (43.8%) EDs reported to have 1 to 4, while 17 (53.1%) had 5 to 9 geriatric-friendly equipment. One (3.1%) ED had 11 elements. Table 2 described the availability, relevance and feasibility of all surveyed equipment.

**Table 2:** Availability, relevance and feasibility of equipment for optimal geriatric care in Flemish emergency departments

	**Does current practice correspond to this standard?** **n (%)**		**How relevant is this standard?** **n (%)**				**How feasible is this standard?** n (%)			
			**Not relevant**	**Rather not relevant**	**Rather** **relevant**	**Very relevant**	**Not feasible**	**Rather not feasible**	**Rather** **feasible**	**Very feasible**
	**Yes**	**No**								
Non-slip socks	12 (37,5)	20 (62,5)	6 (18,8)	6 (18,8)	8 (25)	12 (37,5)	6 (18,8)	9 (28,1)	5 (15,6)	12 (37,5)
Pressure-ulcer reducing mattresses and pillows	27 (84,4)	5 (15,6)	1 (3,2)^a^	1 (3,2)^a^	10 (32,3)^a^	19 (61,3)^a^	1 (3,2)^a^	0 (0)^a^	11 (35,5)^a^	19 (61,3)^a^
Blanket warmer	26 (81,3)	6 (18,8)	3 (9,7)^a^	0 (0)^a^	14 (45,2)^a^	14 (45,2)^a^	2 (6,5)^a^	0 (0)^a^	12 (38,7)^a^	17 (54,8)^a^
Hearing assist devices	2 (6,3)	30 (93,8)	10 (31,3)	14 (43,8)	6 (18,8)	2 (6,3)	21 (65,6)	9 (28,1)	0 (0)	2 (6,3)
Bedside commodes	26 (81,3)	6 (18,8)	1 (3,1)	8 (25)	15 (46,9)	8 (25)	2 (6,3)	1 (3,1)	13 (40,6)	16 (50)
Condom catheters	12 (38,7)^a^	19 (61,3)^a^	3 (9,4)	11 (34,4)	12 (37,5)	6 (18,8)	1 (3,1)	7 (21,9)	11 (34,4)	13 (40,6)
Bedside step stool	7 (21,9)	25 (78,1)	12 (37,5)	5 (15,6)	11 (34,4)	4 (12,5)	9 (28,1)	6 (18,8)	9 (28,1)	8 (25)
Reclining arm chairs	19 (59,4)	13 (40,6)	3 (9,4)	6 (18,8)	14 (43,8)	9 (28,1)	2 (6,3)	10 (31,3)	8 (25)	12 (37,5)
Low beds/ high-low beds	28 (87,5)	4 (12,5)	1 (3,1)	1 (3,1)	7 (21,9)	23 (71,9)	1 (3,1)	0 (0)	7 (21,9)	24 (75)
Cane	2 (6,3)	30 (93,8)	12 (37,5)	10 (31,3)	9 (28,1)	1 (3,1)	8 (25)	6 (18,8)	12 (37,5)	6 (18,8)
4-point cane	1 (3,1)	31 (96,9)	11 (34,4)	9 (28,1)	10 (31,3)	2 (6,3)	9 (28,1)	7 (21,9)	12 (37,5)	4 (12,5)
Walking frame	2 (6,3)	30 (93,8)	10 (31,3)	8 (25)	11 (34,4)	3 (9,4)	9 (29)^a^	8 (25,8)^a^	9 (29)^a^	5 (16,1)^a^
Two wheeled walker	2 (6,3)	30 (93,8)	11 (34,4)	9 (28,1)	10 (31,3)	2 (6,3)	7 (21,9)	9 (28,1)	9 (28,1)	7 (21,9)
Four wheeled walker	2 (6,3)	30 (93,8)	11 (34,4)	10 (31,3)	9 (28,1)	2 (6,3)	8 (28,1)	9 (28,1)	10 (31,3)	5 (15,6)

^a^(*n* = 31)

Four equipment elements were very often available in Flemish EDs: i) pressure-ulcer reducing mattresses and pillows (*n *= 27/32; 84.4%), ii) blanket warmer (*n* = 26/32; 81.3%), iii) bedside commodes (*n* = 26/32; 81.3%) and iv) low beds or high-low beds (*n* = 28/32; 87.5%). The only equipment element that was often available were reclining arm chairs (*n* = 19/32; 59.4%). Each type of walking aid (i.e. cane, four-point cane, walking frame, two wheeled walker and four wheeled walker) was available in only one (3.1%) or two (6.3%) EDs. One ED had both a cane and walker available. Non-slip socks were available in 12 (37.5%) EDs. No region-wide improvement opportunity was identified.

### Physical environment

Out of 11 surveyed physical environment criteria, half of EDs (*n* = 16; 50.0%) reported to have 1 to 4, while the other half had 5 to 9 geriatric-friendly physical environment criteria. Table 3 describes the availability, relevance and feasibility of all surveyed physical environment criteria.

**Table 3:** Availability, relevance and feasibility of physical environment criteria for optimal geriatric care in Flemish emergency departments

	**Does current practice correspond to this standard?** **n (%)**		**How relevant is this standard?** **n (%)**				**How feasible is this standard?** **n (%)**			
			**Not relevant**	**Rather not relevant**	**Rather relevant**	**Very relevant**	**Not feasible**	**Rather not feasible**	**Rather** **feasible**	**Very feasible**
	**Yes**	**No**								
A **separate, physically delineated area** is available to exploit the geriatric emergency care function	2 (6,3)	30 (93,8)	11 (34,4)	9 (28,1)	8 (25)	4 (12,5)	18 (56,3)	8 (25)	6 (18,8)	0 (0)
Easy access to **food and drink**	24 (75)	8 (25)	0 (0)	6 (18,8)	19 (59,4)	7 (21,9)	1 (3,1)	4 (12,5)	19 (59,4)	8 (25)
Ample seating for visitors and family (**at least 2 seats per room**)	27 (84,4)	5 (15,6)	1 (3,2)^a^	3 (9,7)^a^	16 (51,6)^a^	11 (35,5)^a^	2 (6,5)^a^	3 (9,7)^a^	10(32,3)^a^	16 (51,6)^a^
A **large-face analog clock** in each patient room	16 (50)	16 (50)	1 (3,1)	4 (12,5)	13 (40,6)	14 (43,8)	3 (9,4)	1 (3,1)	16 (50)	12 (37,5)
Efforts at **noise reduction** (separate enclosed rooms)	17 (53,1)	15 (46,9)	0 (0)	10 (31,3)	17 (53,1)	5 (15,6)	4 (12,5)	11 (34,4)	13 (40,6)	4 (12,5)
**Enhanced lighting** (e.g. natural light, artificial skylight or window…)	17 (53,1)	15 (46,9)	1 (3,1)	1 (3,1)	21 (65,6)	9 (28,1)	2 (6,3)	11 (34,4)	9 (28,1)	10 (31,3)
**Non-slip floors**	6 (18,8)	26 (81,3)	0 (0)	6 (18,8)	18 (56,3)	8 (25)	5 (15,6)	12 (37,5)	13 (40,6)	2 (6,3)
Adequate **hand rails in sanitary facilities**	24 (75)	8 (25)	0 (0)	0 (0)	13 (40,6)	19 (59,4)	0 (0)	3 (9,4)	10 (31,3)	19 (59,4)
**High-quality signage and way-finding**	22 (68,8)	10 (31,3)	0 (0)	4 (12,5)	12 (37,5)	16 (50)	1 (3,1)	4 (12,5)	12 (37,5)	15 (46,9)
**Wheel-chair accessible toilets**	27 (84,4)	5 (15,6)	0 (0)	0 (0)	10 (31,3)	22 (68,8)	1 (3,1)	1 (3,1)	8 (25)	22 (68,8)
Availability of **raised toilet seats**	11 (34,4)	21 (65,6)	2 (6,3)	1 (3,1)	14 (43,8)	15 (46,9)	3 (9,4)	3 (9,4)	15 (46,9)	11 (34,4)

^a^(*n* = 31)

Two physical environment criteria were very often available on Flemish EDs: i) seating for visitors (i.e. at least two seats per room) (*n* = 27/32; 84.4%) and ii) wheel-chair accessible toilets (*n* = 27/32; 84.4%). Five elements were often available: i) easy access to food and drink (*n* = 24/32; 75%), ii) adequate hand rails in sanitary facilities (*n* = 24/32; 75%), iii) high quality signage and way-finding (*n* = 22/32; 68.8%), iv) enhanced lightning (*n* = 17/32; 53.3%) and v) efforts at noise reduction (*n* = 17/32; 53.1%). Two easy to achieve region-wide improvement opportunities were identified. These included having a large-face and analogue clock in each patient room and availability of raised toilet seats. Availability of non-slip floors was the only identified difficult to achieve region-wide improvement opportunity.

## Discussion

To encourage prioritized incorporation of geriatric emergency guidelines in clinical care, the Geriatric ED Accreditation Program was initiated [8]. The aim of this study was to explore the availability of geriatric-friendly protocols, equipment and physical environment criteria in Flemish EDs and identify related improvement opportunities based on the American Geriatric ED Accreditation Program. The findings of this study should be considered as a context analysis to inspire future initiatives to enhance the care of older ED patients in Flanders.

This study demonstrated that Flemish EDs have taken initiatives on an individual basis to adapt their activities to the needs of older patients. Alongside the benefits for numerous patients, it has also introduced disparities in care between EDs. For example, out of 27 surveyed protocols, one ED reported having 22 protocols available, while one other reported to have none. Although it is unknown to what extent this might impact quality of care and outcomes, the current findings should prompt clinicians and policymakers to determine which protocols, equipment and environmental characteristics should become minimum operational standards in Flemish EDs.

Determining minimum operational standards for geriatric emergency care is not expected to be a straight-forward exercise, as each surveyed standard was already available in at least one Flemish ED. Therefore, it would be appropriate to conduct this exercise with a systematic approach including predefined decision-making rules (i.e. using a Delphi study methodology) [13, 14]. In some respects, the reported availability of surveyed standards can enable this decision-making process. For example, a protocol that was already very often available in Flemish EDs is more likely to become an operational standard. However, excessive focus on already available initiatives could make the minimum standards merely a unification of care. Of course, the purpose should go further and focus especially on what constitutes high quality geriatric emergency care instead of mainly concentrating on what is already available in current EDs.

To guarantee a reflection of high quality geriatric emergency care, operational standards should be defined with an interdisciplinary panel (e.g. ED physicians, ED nurses, geriatricians and geriatric nurses). This might avoid obtaining operational standards with care-related inconsistencies, which are also present in the results of this study. For example, in the current survey, almost 70% of the respondents reported having a standardized fall assessment guideline with follow-up possibilities (*n*= 22/32; 68.8%). Knowing this, it is remarkable that all surveyed walking aids were only available in one or two EDs. Even more, the majority of respondents scored walking aids as not or rather not relevant, while their purpose should be preventing falls during ED stay and avoiding unnecessary admissions among patients with balance difficulties (e.g. some patients with balance difficulties can return home safely if they can use a walking aid correctly during ED stay). As availability of mobility aids is for these reasons one of the few minimum requirements to obtain a geriatric ED accreditation label, it is clear that purchasing walking aids and organizing trainings on how to use them is an important improvement opportunity for Flemish EDs [7].

Another important improvement opportunity for Flemish EDs comprises systematic screening for delirium. This was reported to be available in only three (9,4%) EDs at the moment of the survey (see Additional file 3) and could formally not be labelled a ‘region-wide improvement opportunity’, as it was considered relevant by 68,8% of respondents -which was below the threshold of 75%-. However, despite these findings, systematic delirium screening should be an absolute improvement priority in all EDs. First, because delirium can be an atypical presentation of acute disease (e.g. sepsis) [15–19]. Second, because consequences of undetected delirium have shown to be associated with increased risk of mortality and progressive functional decline [20, 21]. As emergency admissions in vulnerable older patients and the onset of delirium in this population are often medication-related, it is a relevant finding that medication reconciliation was identified a ‘region-wide improvement opportunity’ [15]. However, as both systematic screening for delirium and medication reconciliation were mainly scored as ‘not’ or ‘rather not’ feasible, further research is necessary to improve the use of these protocols in clinical practice. This includes conducting a context analysis including identification of facilitators and barriers that need to be addressed to ensure successful implementation of these protocols in clinical care [22].

A key limitation of this study concerns the generalizability of study findings due to a restricted response rate (i.e. 50.8%). This might have introduced selection bias (e.g. included EDs comprised more public hospitals and less private hospitals, see Additional file 4). Also, results might be biased, as self-report questionnaires were used, allowing for under or over reporting. In addition, another limitation of this study was that the questionnaire only explored those domains of the American Geriatric ED Accreditation Program that were directly related to patient care. As a result, the domains on quality improvement and outcome measurement were not surveyed. Keeping the questionnaire as short as possible alongside assuming that geriatric emergency care initiatives in Flanders were rather in a premature phase led to this choice. In spite of this, these domains play an indispensable role in EDs aiming to provide high-quality geriatric care. Therefore, future research should aim to map geriatric-oriented monitoring initiatives of Flemish EDs. Ideally, consensus is sought on a region-wide set of quality indicators, as well, which can allow benchmarking activities, as was done in the German ‘GeriQ-ED project’ [23].

## Conclusions

To date, Flemish EDs have taken some initiatives on an individual basis to adapt their activities to the needs of older patients. As these have introduced disparities in care between EDs, there is a need to define which geriatric-friendly protocols, equipment and physical environment characteristics should become region-wide minimum operational standards. The methodology and findings of this study can facilitate researchers, clinicians and policy makers in the development process of these operational standards in Belgium and in other countries.

## Supplementary Information

**Additional file 1. **Checklist for reporting of survey studies (CROSS).

**Additional file 2. **Questionnaire.

**Additional file 3. **Availability, relevance and feasibility of few available protocols that were not identified as ‘region-wide improvement opportunities’.

**Additional file 4. **Differences between excluded and included emergency departments.

## Data Availability

The datasets used and/or analysed during the current study are available from the corresponding author on reasonable request.

## Abbreviations

ACEP: American College of Emergency Physicians
ED: Emergency department
EDs: Emergency departments
e.g.: Example given
i.e.: Id est
IQR: Interquartile range

## References

[1] Kahn JH, Magauran BGJ, Olshaker JS (2014). Geriatric Emergency Medicine: principles and practice.

[2] Ellis G, Marshall T, Ritchie C (2014). Comprehensive geriatric assessment in the emergency department. Clin Interv Aging.

[3] Devriendt E, Conroy S, Nickel C, Bellou A, Conroy S (2018). Comprehensive Geriatric Assessment in the Emergency Department. Geriatric Emergency Medicine.

[4] Aminzadeh F, Dalziel WB (2002). Older adults in the emergency department: a systematic review of patterns of use, adverse outcomes, and effectiveness of interventions. Ann Emerg Med.

[5] Carpenter CR, Bromley M, Caterino JM, Chun A, Gerson LW, Greenspan J (2014). Optimal older adult emergency care: introducing multidisciplinary geriatric emergency department guidelines from the American College of Emergency Physicians, American Geriatrics Society, Emergency Nurses Association, and Society for Academic Emergency Medicine. Acad Emerg Med.

[6] Banerjee J, Conroy S, Cooke MW (2013). Quality care for older people with urgent and emergency care needs in UK emergency departments. Emerg Med J.

[7] American College of Emergency Physicians (2021). Geriatric Emergency Department Accreditation Program.

[8] Southerland LT, Lo AX, Biese K, Arendts G, Banerjee J, Hwang U (2020). Concepts in Practice: Geriatric Emergency Departments. Ann Emerg Med.

[9] Heeren P (2021). Effectiveness and improvement opportunities of the 'URGENT' geriatric emergency care model.

[10] Devriendt E, De Brauwer I, Vandersaenen L, Heeren P, Conroy S, Boland B (2017). Geriatric support in the emergency department: a national survey in Belgium. BMC Geriatr.

[11] Sharma  A, Minh Duc NT, Luu Lam Thang T,  Jia Ng S, Said Abbas K (2021). A Consensus-Based Checklist for Reporting of Survey Studies (CROSS). J Gen Intern Med.

[12] Qualtrics. Released 2005. The Qualtrics XM PlatformTM, Version January 2020. Provo, UT, USA: Qualtrics. https://www.qualtrics.com. Accessed 22 August 2022.

[13] Veugelers R, Gaakeer MI, Patka P, Huijsman R (2020). Improving design choices in Delphi studies in medicine: the case of an exemplary physician multi-round panel study with 100% response. BMC Med Res Methodol.

[14] Gaakeer MI, Veugelers R, Patka P, Huijsman R (2019). Minimum operational standards for 24/7 available emergency departments in the Netherlands: a first step taken by emergency physicians using an e-Delphi approach. Eur J Emerg Med.

[15] Han JH, Wilson A, Ely EW (2010). Delirium in the older emergency department patient: a quiet epidemic. Emerg Med Clin North Am.

[16] Calf AH, Pouw MA, van Munster BC, Burgerhof JGM, de Rooij SE, Smidt N (2021). Screening instruments for cognitive impairment in older patients in the Emergency Department: a systematic review and meta-analysis. Age Ageing.

[17] Carpenter CR, Bassett ER, Fischer GM, Shirshekan J, Galvin JE, Morris JC (2011). Four sensitive screening tools to detect cognitive dysfunction in geriatric emergency department patients: brief Alzheimer's Screen, Short Blessed Test, Ottawa 3DY, and the caregiver-completed AD8. Acad Emerg Med.

[18] Inouye SK, van Dyck CH, Alessi CA, Balkin S, Siegal AP, Horwitz RI (1990). Clarifying confusion: the confusion assessment method. A new method for detection of delirium. Ann Intern Med..

[19] Grossmann FF, Hasemann W, Graber A, Bingisser R, Kressig RW, Nickel CH (2014). Screening, detection and management of delirium in the emergency department - a pilot study on the feasibility of a new algorithm for use in older emergency department patients: the modified Confusion Assessment Method for the Emergency Department (mCAM-ED). Scand J Trauma Resusc Emerg Med.

[20] Han JH, Shintani A, Eden S, Morandi A, Solberg LM, Schnell J (2010). Delirium in the emergency department: an independent predictor of death within 6 months. Ann Emerg Med.

[21] Boucher V, Lamontagne ME, Nadeau A, Carmichael PH, Yadav K, Voyer P (2019). Unrecognized Incident Delirium in Older Emergency Department Patients. J Emerg Med.

[22] Eagles D, Cheung WJ, Avlijas T, Yadav K, Ohle R, Taljaard M (2022). Barriers and facilitators to nursing delirium screening in older emergency patients: a qualitative study using the theoretical domains framework. Age Ageing.

[23] Schuster S, Singler K, Lim S, Machner M, Döbler K, Dormann H (2020). Quality indicators for a geriatric emergency care (GeriQ-ED) - an evidence-based delphi consensus approach to improve the care of geriatric patients in the emergency department. Scand J Trauma Resusc Emerg Med.

