# Supplementary material for: A survey on the availability of geriatric-friendly protocols, equipment and physical environment across emergency departments in Flanders, Belgium
Source: BMC Geriatr. 2023 May 3;23:264. doi: 10.1186/s12877-023-03994-z (PMC10155353; doi:10.1186/s12877-023-03994-z)
Supplement: Supplementary file 2 — Additional file 2. Questionnaire. [file 12877_2023_3994_MOESM2_ESM.pdf]

Toepasbaarheid van Amerikaanse accreditatiestandaarden voor geriatrische spoedgevallenzorg in Vlaamse ziekenhuizen

Beste hoofdverpleegkundige,

Recent publiceerde het Amerikaans College van Urgentieartsen een **accreditatieprogramma** voor **geriatrische spoedgevallenzorg** dat focust op zeven domeinen: 1) personeel, 2) educatie, 3) beleid/protocollen, richtlijnen en procedures, 4) kwaliteitsverbetering, 5) uitkomstindicatoren, 6) uitrusting en materialen en 7) accommodatie.

Deze vragenlijst beoogt te peilen naar hoe **toepasbaar** de Amerikaanse accreditatiestandaarden zijn in Vlaamse spoedgevallendiensten. Concreet vragen wij per accreditatiestandaard hoe **relevant** en **haalbaar** deze is voor uw spoedgevallendienst en of de **huidige praktijkvoering** in uw spoedgevallendienst reeds conform deze standaard is. De resultaten van dit onderzoek zullen een bijdrage leveren aan het standaardiseren van geriatrische spoedgevallenzorg in Vlaanderen.

In deze studie worden twee van de zeven domeinen, met name kwaliteitsverbetering en uitkomstindicatoren, buiten beschouwing gelaten, omdat deze verdergaan dan het primaire studiedoel, namelijk toepasbaarheid van zorgstandaarden bevragen. Afhankelijk van de studieresultaten zullen de onderzoekers overwegen om in een vervolgstudie het gebruik en de effecten van zorgstandaarden te bestuderen als onderdeel van kwaliteitsverbetering.

Voordat u de toepasbaarheid van de Amerikaanse accreditatiestandaarden beoordeelt, zult u enkele vragen beantwoorden die peilen naar **persoonlijke gegevens** van uzelf en uw medisch diensthoofd, van wie we vragen om uw antwoorden in te kijken en te valideren. Indien uw mening en die van het medisch diensthoofd zeer verschillend zijn, wordt uw mening genoteerd. U bent vrij om dit meningsverschil achteraan in de vragenlijst als opmerking te noteren. Daarnaast bevat de vragenlijst ook enkele vragen die peilen naar '**kenmerken van het ziekenhuis en de spoedgevallendienst**' en '**de algemene visie op geriatrische spoedgevallenzorg**'.

Gelieve bij gesloten vragen het **vakje** te kleuren of het **cijfer** te omcirkelen van het antwoord dat u wenst aan te duiden. Bij open vragen kan u het **antwoord** op de voorziene **stippellijnen** neerschrijven. Het is voor ons belangrijk dat u de vragen in alle eerlijkheid probeert te beantwoorden. Uw antwoorden zullen **anoniem** gerapporteerd worden. Deze studie werd goedgekeurd door de Ethische Commissie Onderzoek UZ/ KU Leuven.

Wij danken u alvast hartelijk voor uw medewerking!

Met vriendelijke groeten,

Lotte Lombaert, Studente Master in de Verpleegkunde en Vroedkunde, KU Leuven
Petra Janssens, Studente Master in de Verpleegkunde en Vroedkunde, KU Leuven
Pieter Heeren, Doctoraatsstudent Academisch Centrum voor Verpleeg- en Vroedkunde (AccentVV), KU Leuven
Prof. dr. Koen Milisen, Hoogleraar Ouderenzorg, AccentVV, KU Leuven
Prof. dr. Marc Sabbe, Staflid Urgentiegeneeskunde, KU/UZ Leuven

PERSOONLIJKE GEGEVENS (PG)

PERSOONLIJKE GEGEVENS HOOFDVERPLEEGKUNDIGE (PGH)

Geslacht (PGH01)	◯ Vrouw ◯ Man
Leeftijd (PGH02)	………………………… jaren oud
Aantal jaren werkervaring op een spoedgevallendienst (PGH03)	…………………………jaren
Aantal jaren werkervaring als hoofdverpleegkundige op een spoedgevallendienst (PGH04)	…………………………jaren

PERSOONLIJKE GEGEVENS DIENSTHOOFD (PGD)

Geslacht (PGD01)	◯ Vrouw ◯ Man
Leeftijd (PGD02)	………………………… jaren oud
Aantal jaren werkervaring op een spoedgevallendienst (inclusief opleiding)? (PGD03)	…………………………jaren
Aantal jaren werkervaring als diensthoofd van een spoedgevallendienst (PGD04)	…………………………jaren

KENMERKEN VAN HET ZIEKENHUIS EN DE SPOEDGEVALLENDIENST (GZ)

Naam van het ziekenhuis (GZ01)	....
Hoeveel procent van de hospitalisaties in uw ziekenhuis werden in 2018 opgenomen via de spoedgevallendienst? (GZ02)	◯ ....<br>◯ Gegevens zijn niet beschikbaar
De spoedgevallendienst is uitgerust met (GZ03)<br>(*Kruis de aanwezige functies aan*)	◯ Ziekenwagen<br>◯ Paramedisch interventieteam (PIT)<br>◯ Mobiele urgentiegroep (MUG)
Is er een huisartsenwachtpost op de ziekenhuiscampus? (GZ04)	◯ Ja ◯ Neen
Indien ja:<br>*Ligt de huisartsenwachtpost naast (aansluitend) de spoedgevallendienst? (GZ05)*	◯ *Ja* ◯ *Neen*
Hoeveel procent van de spoedverpleegkundigen bezit het diploma 'Bachelor-na-bachelor intensieve zorg en spoedgevallenzorg'? (GZ06)	◯ ....%<br>◯ Gegevens zijn niet beschikbaar

Aantal aanmeldingen op de spoedgevallendienst in 2018 (GZ07)	◯ ....<br>◯ Gegevens zijn niet beschikbaar
Aantal unieke patiënten op de spoedgevallendienst in 2018 (GZ08)	◯ ....<br>◯ Gegevens zijn niet beschikbaar
Aantal unieke patiënten ouder dan 75 jaar op de spoedgevallendienst in 2018 (GZ09)	◯ ....<br>◯ Gegevens zijn niet beschikbaar

Aantal aanmeldingen van personen ouder dan 75 jaar op de spoedgevallendienst in 2018 (GZ10)	◯ ....<br>◯ Gegevens zijn niet beschikbaar
*Aantal hospitalisaties van personen ouder dan 75 jaar vanuit de spoedgevallendienst in 2018 (GZ11)	◯ ....<br>◯ Gegevens zijn niet beschikbaar
*Aantal aanmeldingen van personen ouder dan 75 jaar die werden doorgestuurd naar een secundair ziekenhuis in 2018 (GZ12)	◯ ....<br>◯ Gegevens zijn niet beschikbaar
*Aantal ambulante zorgverstrekkingen van personen ouder dan 75 jaar op de spoedgevallendienst in 2018 (GZ13)	◯ ....<br>◯ Gegevens zijn niet beschikbaar

OPMERKING: De som van de aantallen met * moet gelijk zijn aan het aantal aanmeldingen van personen ouder dan 75 jaar in 2018.

ALGEMENE VISIE OP GERIATRISCHE SPOEDGEVALLENZORG (AV)

In welke mate bent u akkoord met onderstaande stellingen? Bij elke stelling dient u één antwoord aan te duiden.

		Helemaal niet akkoord	Eerder niet akkoord	Geen mening	Eerder akkoord	Helemaal akkoord
AV01	Ouderen hebben een andere klinische aanpak nodig in vergelijking met jongere volwassenen.	○	○	○	○	○
AV02	Ouderen hebben een andere medische aanpak nodig in vergelijking met jongere volwassenen.	○	○	○	○	○
AV03	Ouderen hebben een andere verpleegkundige aanpak nodig in vergelijking met jongere volwassenen.	○	○	○	○	○
AV04	Ouderen hebben aangepast materiaal en infrastructuur nodig in vergelijking met jongere volwassenen.	○	○	○	○	○
AV05	Initiatieven ter verbetering van geriatrische zorg op spoedgevallendiensten zijn noodzakelijk.	○	○	○	○	○
AV06	De zorgcontinuïteit voor ouderen na ontslag van de spoedgevallendienst (bijvoorbeeld naar de eigen woning, een woonzorgcentrum of een geriatrische hospitalisatiedienst) moet verder geoptimaliseerd worden.	○	○	○	○	○

Gelieve voor onderstaande standaarden te scoren hoe relevant en haalbaar u deze vindt en of de huidige praktijkvoering reeds aan deze standaarden voldoet. Omcirkel het cijfer dat u wenst aan te duiden.

STANDAARD	Hoe relevant vindt u deze standaard? (R)				Hoe haalbaar vindt u deze standaard? (H)				Is de huidige praktijkvoering conform deze standaard? (P)	
	Niet relevant	Weinig relevant	Eerder relevant	Heel relevant	Niet haalbaar	Weinig haalbaar	Eerder haalbaar	Heel haalbaar	Ja	Nee
De arts of één van de artsen die de medische permanentie waarneemt, moet in staat zijn om urgente aandoeningen, inclusief atypische presentaties, bij geriatrische patiënten te detecteren en initieel te behandelen. (PN01)	1	2	3	4	1	2	3	4	1	2
De arts of één van de artsen die de medische permanentie waarneemt moet door middel van vooraf opgestelde modaliteiten op z'n minst tijdens daguren (op zowel week- als weekenddagen) beroep kunnen doen op een geriater. (PN02)	1	2	3	4	1	2	3	4	1	2
De verpleegkundige permanentie moet steeds minstens één verpleegkundige ter beschikking hebben die op z'n minst bijscholing heeft gevolgd rond geriatrische zorg op een spoedgevallendienst. (PN03)	1	2	3	4	1	2	3	4	1	2
De medische en verpleegkundige permanentie moeten tijdens daguren (op week- en weekenddagen) beroep kunnen doen op een verpleegkundig casemanager om bij complexe geriatrische patiënten een interdisciplinair plan te maken. Dit interdisciplinair plan documenteert aandachtspunten en acties voor de spoedopname en voorziet continuïteit van zorg en opvolging voor erna. (PN04)	1	2	3	4	1	2	3	4	1	2

De coördinatie en organisatie van de functie ‘geriatrische spoedgevallenzorg’ berusten bij een hiervoor specifiek aangestelde arts. (PN05)	1	2	3	4	1	2	3	4	1	2
Minstens één persoon van het administratief team in het ziekenhuis moet, als deel van zijn/haar portfolio, de functie geriatrische spoedgevallenzorg opvolgen en hierin actief betrokken worden om spoedgevallenzorg voor ouderen te faciliteren (PN06)	1	2	3	4	1	2	3	4	1	2
De spoedgevallendienst moet een patiëntvertegenwoordiger of patiënten-raad aanstellen die op z’n minst maandelijks input kan geven over potentiële verbeteropportuniteiten binnen de functie geriatrische spoedgevallenzorg. (PN07)	1	2	3	4	1	2	3	4	1	2
De artsen van de spoedgevallendienst moeten opleiding volgen over geriatrische zorg. Dit kan gerealiseerd worden via verschillende mogelijkheden, zoals deelname aan een cursus, een bijscholing, een congres/symposium, e-learnings. (ED01)	1	2	3	4	1	2	3	4	1	2
De spoedgevallendienst moet documenteren welke opleidingen de artsen hebben gevolgd. Deze documenten zijn bruikbaar voor accreditatie. (ED02)	1	2	3	4	1	2	3	4	1	2
De verpleegkundigen van de spoedgevallendienst moeten opleiding volgen over geriatrische zorg. Dit kan gerealiseerd worden via verschillende mogelijkheden, zoals deelname aan een cursus, een bijscholing, een congres/symposium, e-learnings. (ED03)	1	2	3	4	1	2	3	4	1	2
De spoedgevallendienst moet documenteren welke opleidingen de verpleegkundigen hebben gevolgd. Deze documenten zijn bruikbaar voor accreditatie. (ED04)	1	2	3	4	1	2	3	4	1	2

Opleidingsinitiatieven voor geriatrische zorg op een spoedgevallendienst moeten focussen op: (*Scoor relevantie, haalbaarheid en huidige praktijk voor ieder domein afzonderlijk*) (ED05)										
1. Atypische presentatie van ziekten (ED05.1)	1	2	3	4	1	2	3	4	1	2
2. Trauma bij ouderen inclusief vallen (ED05.2)	1	2	3	4	1	2	3	4	1	2
3. Cognitieve problemen en storend gedrag bij ouderen (ED05.3)	1	2	3	4	1	2	3	4	1	2
4. Therapieaanpassingen bij ouderen (ED05.4)	1	2	3	4	1	2	3	4	1	2
5. Medicatiemanagement/polyfarmacie (ED05.5)	1	2	3	4	1	2	3	4	1	2
6. Zorgtransities en continuïteit van zorg (ED05.6)	1	2	3	4	1	2	3	4	1	2
7. Effecten van comorbiditeit en polymorbiditeit (ED05.7)	1	2	3	4	1	2	3	4	1	2
8. Palliatieve en terminale zorg (ED05.8)	1	2	3	4	1	2	3	4	1	2

Gelieve voor onderstaande procedures/protocollen te scoren hoe relevant en haalbaar u deze vindt voor de spoedgevallendienst. Gelieve daarnaast ook te scoren of er reeds zo'n procedure aanwezig is in de huidige praktijkvoering. Omcirkel het cijfer dat u wenst aan te duiden.

PROCEDURE	Hoe relevant vindt u deze procedure voor de spoedgevallen-dienst?				Hoe haalbaar vindt u deze procedure voor de spoedgevallen-dienst?				Is er reeds zo'n procedure aanwezig in de huidige praktijkvoering ?	
	Niet relevant	Weinig relevant	Eerder relevant	Heel relevant	Niet haalbaar	Weinig haalbaar	Eerder haalbaar	Heel haalbaar	Ja	Nee
Een procedure die vanaf het triagemoment op de spoedgevallendienst criteria definieert voor toegang tot een specifieke geriatrische benadering (PR01)	1	2	3	4	1	2	3	4	1	2
Een procedure voor gestandaardiseerde deliriumscreening die ook gepaste opvolgingsmogelijkheden voorziet (PR02)	1	2	3	4	1	2	3	4	1	2
Een procedure voor gestandaardiseerde dementiescreening (PR03)	1	2	3	4	1	2	3	4	1	2
Een procedure voor de gestandaardiseerde evaluatie van functionaliteit en functionele achteruitgang die ook gepaste opvolgingsmogelijkheden voorziet (PR04)	1	2	3	4	1	2	3	4	1	2
Een procedure voor de gestandaardiseerde evaluatie van valrisico die ook gepaste opvolgingsmogelijkheden voorziet (PR05)	1	2	3	4	1	2	3	4	1	2
Een procedure voor detectie van ouderenmishandeling die ook gepaste opvolgingsmogelijkheden voorziet (PR06)	1	2	3	4	1	2	3	4	1	2

Een procedure voor medicatie reconciliatie (medicatiehistoriek/-anamnese en -ontslagplan) in samenspraak met een klinisch apotheker (PR07)	1	2	3	4	1	2	3	4	1	2
Een procedure om mogelijks ongepast geneesmiddelengebruik te minimaliseren (PR08)	1	2	3	4	1	2	3	4	1	2
Een procedure voor de behandeling van pijn bij geriatrische patiënten (PR09)	1	2	3	4	1	2	3	4	1	2
Een procedure voor een consult palliatieve zorg op de spoedgevallendienst (PR10)	1	2	3	4	1	2	3	4	1	2
Een procedure voor een consult (geronto)psychiatrie op de spoedgevallendienst (PR11)	1	2	3	4	1	2	3	4	1	2
Gebruik van een protocol voor de work-up en initiële behandeling van minstens drie frequent voorkomende spoedpresentaties van ouderen (bijvoorbeeld delirium, heupfractuur, syncope, sepsis, myocardinfarct, beroerte…). Deze protocollen standaardiseren niet alleen de aanvraag van technische onderzoeken (inclusief laboratoriumbepalingen), maar bevatten ook behandelplannen (inclusief medicatiebeleid dat is aangepast aan ouderen). (PR12)	1	2	3	4	1	2	3	4	1	2
Een procedure om het gebruik van urinekatheters te standaardiseren en te minimaliseren. (PR13)	1	2	3	4	1	2	3	4	1	2
Een procedure om de niets per os (NPO) status te minimaliseren en de toegang tot aangepast voedsel en drank te bevorderen (PR14)	1	2	3	4	1	2	3	4	1	2
Een procedure die bewegen en mobiliteit promoot (PR15)	1	2	3	4	1	2	3	4	1	2
Een procedure voor de inschakeling van vrijwilligers (PR16)	1	2	3	4	1	2	3	4	1	2

Een procedure om tegemoet te komen aan specifieke communicatienoden van oudere personen die naar huis ontslagen worden. (Ontslagbrief bevat groot lettertype, lekentaal, duidelijk plan voor opvolging, samenvatting van wat met de patiënt besproken is, etc.) (PR17)	1	2	3	4	1	2	3	4	1	2
Een procedure om de huisarts in te lichten (kennisgeving van opname en verloop) (PR18)	1	2	3	4	1	2	3	4	1	2
Een procedure om ontslag naar een residentiële instelling te faciliteren (PR19)	1	2	3	4	1	2	3	4	1	2
Een procedure om het gebruik van fysieke fixatiemaatregelen tot een absoluut minimum te beperken (PR20)	1	2	3	4	1	2	3	4	1	2
Een procedure voor gestandaardiseerde toegang tot programma’s in het geriatrisch dagziekenhuis (geriatrische evaluatie en oppuntstelling, valkliniek, geheugenkliniek, …) (PR21)	1	2	3	4	1	2	3	4	1	2
Een procedure voor opvolging na ontslag van de spoedgevallendienst (telefonische opvolging, telegeneeskunde…) (PR22)	1	2	3	4	1	2	3	4	1	2
Een procedure voor toegang tot vervoersdiensten die patiënten terugbrengen naar de woonplaats (PR23)	1	2	3	4	1	2	3	4	1	2
Een procedure voor vlotte toegang tot korte- en langetermijn-revalidatiediensten zowel binnen als buiten het ziekenhuis (PR24)	1	2	3	4	1	2	3	4	1	2
Een procedure om de functionaliteit en veiligheid van een patiënt in de eigen woning ter plaatse te (laten) beoordelen (outreach) (PR25)	1	2	3	4	1	2	3	4	1	2
Procedures voor actieve samenwerking met paramedische diensten in de omgeving van het ziekenhuis (Bijvoorbeeld: verhuur van hulpmiddelen/valalarmsystemen, thuisverpleging, apothekers…) (PR26)	1	2	3	4	1	2	3	4	1	2

Een procedure om bewoners van een residentiële instelling ter plaatse te evalueren en indien mogelijk te behandelen (outreach). De doelstelling van dit protocol is om de zorgkwaliteit ter plaatse te verbeteren en het aantal transfers naar de spoedgevallendienst te reduceren. (PR27)	1	2	3	4	1	2	3	4	1	2

Gelieve voor onderstaande loophulpmiddelen te scoren hoe relevant en haalbaar u het vindt om deze op de spoedgevallendienst te gebruiken en of deze reeds aanwezig zijn in de huidige praktijkvoering. Omcirkel het cijfer dat u wenst aan te duiden.

	**Hoe relevant vindt u dit loophulpmiddel op de spoedgevallen-dienst?**				**Hoe haalbaar is het om dit loophulpmiddel op de spoedgevallen-dienst te hebben?**				**Is dit loophulpmiddel reeds aanwezig in de huidige praktijkvoering ?**	
**LOOPHULPMIDDEL**	Niet relevant	Weinig relevant	Eerder relevant	Heel relevant	Niet haalbaar	Weinig haalbaar	Eerder haalbaar	Heel haalbaar	Ja	Nee
Wandelstok (BN01)	1	2	3	4	1	2	3	4	1	2
4-punts wandelstok (BN02)	1	2	3	4	1	2	3	4	1	2
Loopkader (BN03)	1	2	3	4	1	2	3	4	1	2
2-wiel rollator (BN04)	1	2	3	4	1	2	3	4	1	2
4-wiel rollator (BN05)	1	2	3	4	1	2	3	4	1	2
Andere (BN06) …………………………………………………………………………………	1	2	3	4	1	2	3	4	1	2

Gelieve voor onderstaande uitrustingselementen te scoren hoe relevant en haalbaar u het vindt om deze op de spoedgevallendienst te gebruiken en of deze reeds aanwezig zijn in de huidige praktijkvoering. Omcirkel het cijfer dat u wenst aan te duiden.

UITRUSTINGSELEMENT	Hoe relevant vindt u dit uitrustingselement op de spoedgevallen-dienst?				Hoe haalbaar is het om dit uitrustingselement op de spoedgevallen-dienst te hebben?				Is dit uitrustings-element reeds aanwezig in de huidige praktijkvoering ?	
	Niet relevant	Weinig relevant	Eerder relevant	Heel relevant	Niet haalbaar	Weinig haalbaar	Eerder haalbaar	Heel haalbaar	Ja	Nee
Anti-slip kousen (BN07)	1	2	3	4	1	2	3	4	1	2
Anti-decubitus matras en kussens (BN08)	1	2	3	4	1	2	3	4	1	2
Verwarmingsdeken (BN09)	1	2	3	4	1	2	3	4	1	2
Hoorapparaten (BN10)	1	2	3	4	1	2	3	4	1	2
Nachtkastje (met bedtafel) (BN11)	1	2	3	4	1	2	3	4	1	2
Condoomkatheters (BN12)	1	2	3	4	1	2	3	4	1	2
Opstapbankje bij ieder bed (BN13)	1	2	3	4	1	2	3	4	1	2
Fauteuil met verstelbare rugleuning (BN14)	1	2	3	4	1	2	3	4	1	2
Lage bedden/hoog-laag bedden (BN15)	1	2	3	4	1	2	3	4	1	2
Andere (BN16) …………………………………………	1	2	3	4	1	2	3	4	1	2

Gelieve voor onderstaande accommodatiecriteria te scoren hoe relevant en haalbaar u het vindt om deze op de spoedgevallendienst toe te passen en of deze reeds van toepassing zijn in de huidige praktijkvoering. Omcirkel het cijfer dat u wenst aan te duiden.

ACCOMMODATIECRITERIUM	Hoe relevant vindt u dit criterium op de spoedgevallendienst?				Hoe haalbaar vindt u dit criterium op de spoedgevallendienst?				Is dit criterium reeds van toepassing in de huidige praktijkvoering ?	
	Niet relevant	Weinig relevant	Eerder relevant	Heel relevant	Niet haalbaar	Weinig haalbaar	Eerder haalbaar	Heel haalbaar	Ja	Nee
Er is een aparte, fysiek afgebakende ruimte beschikbaar om de functie geriatrische spoedgevallenzorg te exploiteren. (FO01)	1	2	3	4	1	2	3	4	1	2
Voedsel en drank moeten makkelijk te verkrijgen zijn. (FO02)	1	2	3	4	1	2	3	4	1	2
Er dienen per bed twee stoelen beschikbaar te zijn om bezoek te faciliteren (FO03)	1	2	3	4	1	2	3	4	1	2
Er dient in iedere patiëntenkamer een grote analoge klok aanwezig te zijn (FO04)	1	2	3	4	1	2	3	4	1	2
Er moeten inspanningen gedaan worden om lawaai te verminderen (bijvoorbeeld afsluitbare ruimtes) (FO05)	1	2	3	4	1	2	3	4	1	2
Er moet aangepaste verlichting aanwezig zijn (bijvoorbeeld natuurlijk licht, dimbare lampen…) (FO06)	1	2	3	4	1	2	3	4	1	2
Er moet een antislip vloer aanwezig zijn (FO07)	1	2	3	4	1	2	3	4	1	2

In sanitaire voorzieningen (de badkamer en de toiletten) voor patiënten moeten handvaten aanwezig zijn (FO08)	1	2	3	4	1	2	3	4	1	2
Er dient een duidelijke en kwaliteitsvolle bewegwijzering te zijn. (FO09)	1	2	3	4	1	2	3	4	1	2
Er moeten rolstoeltoegankelijke toiletten zijn. (FO10)	1	2	3	4	1	2	3	4	1	2
Er moeten verhoogde toiletzittingen beschikbaar zijn. (FO11)	1	2	3	4	1	2	3	4	1	2
Andere (FO12) ………………………………………………………………………………………………	1	2	3	4	1	2	3	4	1	2

BEZETTING (PN08)

Gelieve in onderstaande tabellen per stelling de **HUIDIGE beschikbaarheid** van de verschillende disciplines op de dienst spoedgevallen op verschillende tijdsmomenten (dag en nacht tijdens week- en weekenddagen) aan te duiden.

Beschikbaarheid van de geriater (PN08.G)

	Weekdag (WD)		Weekenddag (WE)	
	Dag	Nacht	Dag	Nacht
De geriater is **telefonisch consulteerbaar** voor geriatrische patiënten op de spoedgevallendienst (PN08.G01)	◯ Ja ◯ Nee	◯ Ja ◯ Nee	◯ Ja ◯ Nee	◯ Ja ◯ Nee
De geriater is na telefonisch contact **beschikbaar aan bed voor specifieke casussen** (PN08.G02)	◯ Ja ◯ Nee	◯ Ja ◯ Nee	◯ Ja ◯ Nee	◯ Ja ◯ Nee
De geriater is na telefonisch contact **beschikbaar aan bed voor alle casussen** (PN08.G03)	◯ Ja ◯ Nee	◯ Ja ◯ Nee	◯ Ja ◯ Nee	◯ Ja ◯ Nee
De geriater is op **afgesproken momenten aanwezig** op de spoedgevallendienst (PN08.G04)	◯ Ja ◯ Nee	◯ Ja ◯ Nee	◯ Ja ◯ Nee	◯ Ja ◯ Nee
De geriater is **continu aanwezig** op de spoedgevallendienst (PN08.G05)	◯ Ja ◯ Nee	◯ Ja ◯ Nee	◯ Ja ◯ Nee	◯ Ja ◯ Nee

Beschikbaarheid van de sociale dienst (PN08.S)

	Weekdag		Weekenddag	
	Dag	Nacht	Dag	Nacht
De sociale dienst is **telefonisch consulteerbaar** voor geriatrische patiënten op de spoedgevallendienst (PN08.S01)	◯ Ja ◯ Nee	◯ Ja ◯ Nee	◯ Ja ◯ Nee	◯ Ja ◯ Nee
De sociale dienst is na telefonisch contact **beschikbaar aan bed voor specifieke casussen** (PN08.S02)	◯ Ja ◯ Nee	◯ Ja ◯ Nee	◯ Ja ◯ Nee	◯ Ja ◯ Nee
De sociale dienst is na telefonisch contact **beschikbaar aan bed voor alle casussen** (PN08.S03)	◯ Ja ◯ Nee	◯ Ja ◯ Nee	◯ Ja ◯ Nee	◯ Ja ◯ Nee
De sociale dienst is op **afgesproken momenten aanwezig** op de spoedgevallendienst (PN08.S04)	◯ Ja ◯ Nee	◯ Ja ◯ Nee	◯ Ja ◯ Nee	◯ Ja ◯ Nee
De sociale dienst is **continu aanwezig** op de spoedgevallendienst (PN08.S05)	◯ Ja ◯ Nee	◯ Ja ◯ Nee	◯ Ja ◯ Nee	◯ Ja ◯ Nee

Beschikbaarheid van een verpleegkundig casemanager. (Dit kan een verpleegkundige van de interne liaison geriatrie zijn) (PN08.V)

	Weekdag		Weekenddag	
	Dag	Nacht	Dag	Nacht
De verpleegkundig casemanager is **telefonisch consulteerbaar** voor geriatrische patiënten op de spoedgevallendienst (PN08.V01)	◯ Ja ◯ Nee	◯ Ja ◯ Nee	◯ Ja ◯ Nee	◯ Ja ◯ Nee
De verpleegkundig casemanager is na telefonisch contact **beschikbaar aan bed voor specifieke casussen** (PN08.V02)	◯ Ja ◯ Nee	◯ Ja ◯ Nee	◯ Ja ◯ Nee	◯ Ja ◯ Nee
De verpleegkundig casemanager is na telefonisch contact **beschikbaar aan bed voor alle casussen** (PN08.V03)	◯ Ja ◯ Nee	◯ Ja ◯ Nee	◯ Ja ◯ Nee	◯ Ja ◯ Nee
De verpleegkundig casemanager is op **afgesproken momenten aanwezig** op de spoedgevallendienst (PN08.V04)	◯ Ja ◯ Nee	◯ Ja ◯ Nee	◯ Ja ◯ Nee	◯ Ja ◯ Nee
De verpleegkundig casemanager is **continu aanwezig** op de spoedgevallendienst (PN08.V05)	◯ Ja ◯ Nee	◯ Ja ◯ Nee	◯ Ja ◯ Nee	◯ Ja ◯ Nee

Beschikbaarheid van een ergotherapeut. (UITSLUITEND in de functie van ergotherapeut. NIET als lid van de interne liaison geriatrie.) (PN08.E)

	Weekdag		Weekenddag	
	Dag	Nacht	Dag	Nacht
De ergotherapeut is **telefonisch consulteerbaar** voor geriatrische patiënten op de spoedgevallendienst (PN08.E01)	◯ Ja ◯ Nee	◯ Ja ◯ Nee	◯ Ja ◯ Nee	◯ Ja ◯ Nee
De ergotherapeut is na telefonisch contact **beschikbaar aan bed voor specifieke casussen** (PN08.E02)	◯ Ja ◯ Nee	◯ Ja ◯ Nee	◯ Ja ◯ Nee	◯ Ja ◯ Nee
De ergotherapeut is na telefonisch contact **beschikbaar aan bed voor alle casussen** (PN08.E03)	◯ Ja ◯ Nee	◯ Ja ◯ Nee	◯ Ja ◯ Nee	◯ Ja ◯ Nee
De ergotherapeut is op **afgesproken momenten aanwezig** op de spoedgevallendienst (PN08.E04)	◯ Ja ◯ Nee	◯ Ja ◯ Nee	◯ Ja ◯ Nee	◯ Ja ◯ Nee
De ergotherapeut is **continu aanwezig** op de spoedgevallendienst (PN08.E05)	◯ Ja ◯ Nee	◯ Ja ◯ Nee	◯ Ja ◯ Nee	◯ Ja ◯ Nee

Beschikbaarheid van de kinesitherapeut. (UITSLUITEND in de functie van kinesitherapeut. NIET als lid van de interne liaison geriatrie.) (PN08.K)

	Weekdag		Weekenddag	
	Dag	Nacht	Dag	Nacht
De kinesitherapeut is **telefonisch consulteerbaar** voor geriatrische patiënten op de spoedgevallendienst (PN08.K01)	◯ Ja<br>◯ Nee	◯ Ja<br>◯ Nee	◯ Ja<br>◯ Nee	◯ Ja<br>◯ Nee
De kinesitherapeut is na telefonisch contact **beschikbaar aan bed voor specifieke casussen** (PN08.K02)	◯ Ja<br>◯ Nee	◯ Ja<br>◯ Nee	◯ Ja<br>◯ Nee	◯ Ja<br>◯ Nee
De kinesitherapeut is na telefonisch contact **beschikbaar aan bed voor alle casussen** (PN08.K03)	◯ Ja<br>◯ Nee	◯ Ja<br>◯ Nee	◯ Ja<br>◯ Nee	◯ Ja<br>◯ Nee
De kinesitherapeut is op **afgesproken momenten aanwezig** op de spoedgevallendienst (PN08.K04)	◯ Ja<br>◯ Nee	◯ Ja<br>◯ Nee	◯ Ja<br>◯ Nee	◯ Ja<br>◯ Nee
De kinesitherapeut is **continu aanwezig** op de spoedgevallendienst (PN08.K05)	◯ Ja<br>◯ Nee	◯ Ja<br>◯ Nee	◯ Ja<br>◯ Nee	◯ Ja<br>◯ Nee

Beschikbaarheid van de klinisch apotheker (PN08.Z)

	Weekdag		Weekenddag	
	Dag	Nacht	Dag	Nacht
De klinisch apotheker is **telefonisch consulteerbaar** voor geriatrische patiënten op de spoedgevallendienst (PN08.Z01)	◯ Ja<br>◯ Nee	◯ Ja<br>◯ Nee	◯ Ja<br>◯ Nee	◯ Ja<br>◯ Nee
De klinisch apotheker is na telefonisch contact **beschikbaar aan bed voor specifieke casussen** (PN08.Z02)	◯ Ja<br>◯ Nee	◯ Ja<br>◯ Nee	◯ Ja<br>◯ Nee	◯ Ja<br>◯ Nee
De klinisch apotheker is na telefonisch contact **beschikbaar aan bed voor alle casussen** (PN08.Z03)	◯ Ja<br>◯ Nee	◯ Ja<br>◯ Nee	◯ Ja<br>◯ Nee	◯ Ja<br>◯ Nee
De klinisch apotheker is op **afgesproken momenten aanwezig** op de spoedgevallendienst (PN08.Z04)	◯ Ja<br>◯ Nee	◯ Ja<br>◯ Nee	◯ Ja<br>◯ Nee	◯ Ja<br>◯ Nee
De klinisch apotheker is **continu aanwezig** op de spoedgevallendienst (PN08.Z05)	◯ Ja<br>◯ Nee	◯ Ja<br>◯ Nee	◯ Ja<br>◯ Nee	◯ Ja<br>◯ Nee

Beschikbaarheid van de diëtist (PN08.D)

	Weekdag		Weekenddag	
	Dag	Nacht	Dag	Nacht
De diëtist is **telefonisch consulteerbaar** voor geriatrische patiënten op de spoedgevallendienst (PN08.D01)	◯ Ja ◯ Nee	◯ Ja ◯ Nee	◯ Ja ◯ Nee	◯ Ja ◯ Nee
De diëtist is na telefonisch contact **beschikbaar aan bed voor specifieke casussen** (PN08.D02)	◯ Ja ◯ Nee	◯ Ja ◯ Nee	◯ Ja ◯ Nee	◯ Ja ◯ Nee
De diëtist is na telefonisch contact **beschikbaar aan bed voor alle casussen** (PN08.D03)	◯ Ja ◯ Nee	◯ Ja ◯ Nee	◯ Ja ◯ Nee	◯ Ja ◯ Nee
De diëtist is op **afgesproken momenten aanwezig** op de spoedgevallendienst (PN08.D04)	◯ Ja ◯ Nee	◯ Ja ◯ Nee	◯ Ja ◯ Nee	◯ Ja ◯ Nee
De diëtist is **continu aanwezig** op de spoedgevallendienst (PN08.D05)	◯ Ja ◯ Nee	◯ Ja ◯ Nee	◯ Ja ◯ Nee	◯ Ja ◯ Nee

Gelieve in onderstaande tabel per stelling de HUIDIGE beschikbaarheid van het intern liaison team geriatrie (ILG)* op de dienst spoedgevallen op verschillende tijdsmomenten (dag en nacht tijdens week- en weekenddagen) aan te duiden. (PN08.I)

**Synoniemen van de term 'intern liaison team geriatrie' zijn geriatrisch consultatie team, geriatrisch support team,…*

	Weekdag		Weekenddag	
	Dag	Nacht	Dag	Nacht
De ILG-medewerker is **telefonisch consulteerbaar** voor geriatrische patiënten op de spoedgevallendienst (PN08.I01)	◯ Ja ◯ Nee	◯ Ja ◯ Nee	◯ Ja ◯ Nee	◯ Ja ◯ Nee
De ILG-medewerker is na telefonisch contact **beschikbaar aan bed voor specifieke casussen** (PN08.I02)	◯ Ja ◯ Nee	◯ Ja ◯ Nee	◯ Ja ◯ Nee	◯ Ja ◯ Nee
De ILG-medewerker is na telefonisch contact **beschikbaar aan bed voor alle casussen** (PN08.I03)	◯ Ja ◯ Nee	◯ Ja ◯ Nee	◯ Ja ◯ Nee	◯ Ja ◯ Nee
De ILG-medewerker is op **afgesproken momenten aanwezig** op de spoedgevallendienst (PN08.I04)	◯ Ja ◯ Nee	◯ Ja ◯ Nee	◯ Ja ◯ Nee	◯ Ja ◯ Nee
De ILG-medewerker is **continu aanwezig** op de spoedgevallendienst (PN08.I05)	◯ Ja ◯ Nee	◯ Ja ◯ Nee	◯ Ja ◯ Nee	◯ Ja ◯ Nee

Gelieve aan te duiden welke disciplines MOMENTEEL als lid van de interne liaison geriatrie (ILG) casemanagement doen op de spoedgevallendienst (PN08.I)

DISCIPLINE	ILG-medewerker op spoedgevallendienst?
Geriater (PN08.I06)	◯ Ja ◯ Nee
Sociaal assistent (PN08.I07)	◯ Ja ◯ Nee
Verpleegkundig casemanager (PN08.I08)	◯ Ja ◯ Nee
Ergotherapeut (PN08.I09)	◯ Ja ◯ Nee
Kinesitherapeut (PN08.I10)	◯ Ja ◯ Nee
Ziekenhuisapotheker (PN08.I11)	◯ Ja ◯ Nee
Diëtist (PN08.I12)	◯ Ja ◯ Nee
Andere (PN08.I13) …………………………………………………………………………	◯ Ja ◯ Nee

Gelieve voor onderstaande disciplines te scoren hoe relevant en haalbaar u hun aanwezigheid op de spoedgevallendienst inschat. Omcirkel het cijfer dat u wenst aan te duiden.

	Hoe relevant vindt de aanwezigheid van deze discipline op de spoedgevallendienst?				Hoe haalbaar vindt u het om deze discipline aanwezig te hebben op de spoedgevallen-dienst?			
DISCIPLINE	Niet relevant	Weinig relevant	Eerder relevant	Heel relevant	Niet haalbaar	Weinig haalbaar	Eerder haalbaar	Heel haalbaar
Geriater (PN09)	1	2	3	4	1	2	3	4
Sociaal assistent (PN10)	1	2	3	4	1	2	3	4
Verpleegkundig casemanager (PN11)	1	2	3	4	1	2	3	4
Ergotherapeut (PN12)	1	2	3	4	1	2	3	4
Kinesitherapeut (PN13)	1	2	3	4	1	2	3	4
Ziekenhuisapotheker (PN14)	1	2	3	4	1	2	3	4
Diëtist (PN15)	1	2	3	4	1	2	3	4
Andere (PN16) …………………………………………………………………………	1	2	3	4	1	2	3	4

AFSLUITENDE VRAGEN (AFV)

Heeft u nog suggesties/bemerkingen die u wenst te delen met de onderzoekers (AFV01)

Ik wens graag op de hoogte gebracht te worden van de studieresultaten en bijgevolg een digitale versie van de masterproef te ontvangen (AFV02)	◯ Ja<br>◯ Nee
