# Supplementary material for: A survey on the availability of geriatric-friendly protocols, equipment and physical environment across emergency departments in Flanders, Belgium
Source: BMC Geriatr. 2023 May 3;23:264. doi: 10.1186/s12877-023-03994-z (PMC10155353; doi:10.1186/s12877-023-03994-z)
Supplement: Supplementary file 3 — Additional file 3. Availability, relevance and feasibility of few available protocols that were not identified as ‘region-wide improvement opportunities’. [file 12877_2023_3994_MOESM3_ESM.docx]

**ADDITIONAL FILE 3. Availability, relevance and feasibility of few available protocols that were not identified as ‘region-wide improvement opportunities’**

|  | **Does current practice correspond to this standard?**  **Rather relevant**  **n (%)** | | **How relevant is this standard?**  **n (%)** | | | | **How feasible is this standard?**  **n (%)** | | | |
| --- | --- | --- | --- | --- | --- | --- | --- | --- | --- | --- |
|  |  |  | **Not relevant** | **Rather not relevant** | **Rather relevant** | **Very relevant** | **Not feasible** | **Rather not feasible** | **Rather**  **feasible** | **Very feasible** |
|  | **Yes** | **No** |  |  |  |  |  |  |  |  |
| A **standardized dementia screening** process | 2  (6,3) | 30 (93,8) | 7 (21,9) | 13 (40,6) | 8  (25) | 4 (12,5) | 8  (25) | 20 (62,5) | 1  (3,1) | 3  (9,4) |
| A guideline for **standardized assessment of function and functional decline** with appropriate follow-up | 1  (3,1) | 31 (96,9) | 6 (18,8) | 14 (43,8) | 9 (28,1) | 3  (9,4) | 8  (25) | 19 (59,4) | 3  (9,4) | 2  (6,3) |
| A guideline for to minimize the use of **potentially inappropriate medications** | 4  (12,5) | 28 (87,5) | 3  (9,4) | 10 (31,3) | 8  (25) | 11 (34,4) | 3  (9,4) | 17 (53,1) | 7 (21,9) | 5 (15,6) |
| A guideline for accessing **palliative care consultation** in the ED | 16  (50) | 16  (50) | 3  (9,4) | 7 (21,9) | 13 (40,6) | 9 (28,1) | 3  (9,4) | 10 (31,3) | 8  (25) | 11 (34,4) |
| A guideline for accessing **Geriatric Psychiatry consultation** in the ED | 10 (31,3) | 22 (68,8) | 5 (15,6) | 8  (25) | 14 (43,8) | 5 (15,6) | 8  (25) | 11 (34,4) | 7 (21,9) | 6 (18,8) |
| A guideline to **promote mobility** | 4  (12,5) | 28 (87,5) | 6 (18,8) | 10 (31,3) | 9 (28,1) | 7 (21,9) | 6 (18,8) | 18 (56,3) | 4 (12,5) | 4 (12,5) |
| A guideline to guide the use of **volunteer engagement** | 9  (28,1) | 23 (71,9) | 4 (12,5) | 5 (15,6) | 11 (34,4) | 12 (37,5) | 5 (15,6) | 9 (28,1) | 10 (31,3) | 8  (25) |
| A **standardized discharge guideline** for patients discharged home that addresses age-specific communication needs (large-font, lay person’s language, clear follow-up plan, evidence of patient communication) | 2  (6,3) | 30 (93,8) | 3  (9,4) | 6 (18,8) | 14 (43,8) | 9 (28,1) | 2  (6,3) | 10 (31,3) | 15 (46,9) | 5  (16) |
| A **standardized delirium screening** guideline with appropriate follow-up | 3  (9,4) | 29 (90,6) | 2  (6,3) | 8  (25) | 15 (46,9) | 7 (21,9) | 2  (6,3) | 17 (53,1) | 9 (28,1) | 4 (12,5) |
| A guideline for **post-discharge follow up** (phone, telemedicine, other) | 3  (9,4) | 29 (90,6) | 7 (21,9) | 8  (25) | 11 (34,4) | 6 (18,8) | 9 (28,1) | 13 (40,6) | 4 (12,5) | 6 (18,8) |
| A pathway program providing **easy access to short- or long-term rehabilitation services**, including inpatient | 7  (21,9) | 25 (78,1) | 8  (25) | 7 (21,9) | 9 (28,1) | 8  (25) | 8  (25) | 14 (43,8) | 5 (15,6) | 5 (15,6) |
| Access to an **outreach program providing home assessment** of function and safety | 2  (6,3) | 30 (93,8) | 8  (25) | 12 (37,5) | 5 (15,6) | 7 (21,9) | 10 (31,3) | 15 (46,9) | 2  (6,3) | 5 (15,6) |
| Access to and an active relationship with **community paramedicine follow up services** | 7  (21,9) | 25 (78,1) | 7 (21,9) | 7 (21,9) | 13 (40,6) | 5 (15,6) | 7 (21,9) | 14 (43,8) | 7 (21,9) | 4 (12,5) |
| An **outreach program to residential care homes** to enhance quality of care and of ED transfers | 3  (9,4) | 29 (90,6) | 4 (12,5) | 5 (15,6) | 12 (37,5) | 11 (34,4) | 6 (18,8) | 14 (43,8) | 7 (21,9) | 5 (15,6) |
