# Supplementary material for: A survey on the availability of geriatric-friendly protocols, equipment and physical environment across emergency departments in Flanders, Belgium
Source: BMC Geriatr. 2023 May 3;23:264. doi: 10.1186/s12877-023-03994-z (PMC10155353; doi:10.1186/s12877-023-03994-z)
Supplement: Supplementary file 4 — Additional file 4. Differences between excluded and included emergency departments. [file 12877_2023_3994_MOESM4_ESM.docx]

**ADDITIONAL FILE 4. Differences between excluded and included emergency departments**

| **Hospital type*** | **Excluded emergency departments** | **Included emergency departments** | **Total** |
| --- | --- | --- | --- |
| - General hospital | 28 | 22 | 50 |
| - General hospital with university character | 2 | 7 | 9 |
| - University hospital | 1 | 3 | 4 |
| **Total** | 31 | 32 | 63 |

***Chi-Square Test (2-sided): p=0,106.**

| **Number of beds^£^** | **Excluded emergency departments** | **Included emergency departments** | **Total** |
| --- | --- | --- | --- |
| - ≤250 | 9 | 8 | 17 |
| - 251-450 | 14 | 12 | 26 |
| - > 450 | 8 | 12 | 20 |
| **Total** | 31 | 32 | 63 |

**^£^Chi Square Test (2-sided): p=0,607.**

| **Hospital status^$^** | **Excluded emergency departments** | **Included emergency departments** | **Total** |
| --- | --- | --- | --- |
| - Public | 0 | 8 | 8 |
| - Private | 31 | 24 | 55 |
| **Total** | 31 | 32 | 63 |

**^$^Chi Square Test (2-sided): p=0,003 | Fisher’s Exact Test (2-sided): p=0,005.**
